# Characterizing suicidal intent among suicidal adolescents: a systematic review

**DOI:** 10.1186/s13034-025-01019-8

**Published:** 2026-01-02

**Authors:** Romain Sibut, Clara Robert, Margaux Leboulleux, Jonathan Lachal

**Affiliations:** 1 Service de Psychiatrie de l’Enfant et de l’Adolescent, CHU de Clermont-Ferrand, 58 rue Montalembert, Clermont-Ferrand, 63000 France; 2 Université Clermont Auvergne, CHU Clermont-Ferrand, CNRS, IP, UMR 6602, Clermont-Ferrand, France; 3 CESP, Team DevPsy, Université Paris-Saclay, UVSQ, Inserm, Villejuif, 94807 France

**Keywords:** Suicidal intent, Suicide attempt, Adolescent, Young, Suicidal intent scale

## Abstract

**Background:**

Suicidal intent contributes both to the assessment of suicide risk and to long-term prognosis in adults. Although suicidal intent is a key component in suicide risk assessment, its specific features and clinical implications in adolescents after a suicide attempt remain underexplored in the literature. However, it could represent an improvement in the assessment and prognosis of adolescent suicidal crisis. The aim of this study is to describe how suicidal intent manifests in adolescents after a suicide attempt, and how it relates to associated clinical and contextual characteristics.

**Method:**

We conducted a systematic review assessing suicidal intent in adolescents after a suicide attempt adhering to PRISMA guidelines. Five databases were searched up to September 2023. Seventeen studies met the inclusion criteria. We excluded studies focusing solely on suicidal ideation or on non-suicidal self-injury. Data were extracted and synthesized narratively. Study quality was assessed using standard tools.

**Results:**

Several studies suggest that suicidal intent may be more frequently reported in older adolescents, with a significant difference before and after the age of 16. High suicidal intent seems to be more frequently linked to internalized disorders. While suicidal intent does not appear directly linked to the lethality of the attempt, the highest level of suicidal intent reported across previous attempts may represent a prognostic marker for later suicide mortality.

**Conclusion:**

It seems essential to refine existing assessment tools or develop new ones specifically adapted to adolescents, in order to assess suicidal intent while taking into account the specificities of the adolescent population. This would help optimize interventions and support for both the patient and their family.

**Supplementary Information:**

The online version contains supplementary material available at 10.1186/s13034-025-01019-8.

## Introduction

Suicide is a main leading cause of death among youths aged 10–24 years [1]. Numerous studies have focused on identifying risk factors for suicidal crisis and death, with the aim of preventing suicide attempts (SA) and recurrences [1]. Suicidal crisis is defined as a state of acute psychological distress with suicidal ideation, diminished coping strategies, and an increased risk of self-harm or SA [2]. Suicide risk assessment is widely recommended in clinical practice, particularly for the identification and management of suicidal patients [3–5]. However, the number of patients admitted to emergency departments for suicidal crises and SA has been rising over the past ten years [1], and especially since the COVID-19 pandemic [6, 7]. In this context, further exploration of suicidal mechanisms and the development of complementary tools seem essential.

A history of SA is one of the main risk factors for suicide completion and recurrence [8, 9]. It therefore seems of interest to take a closer look at the characteristics of SA in order to refine our understanding of their prognostic value [10]. Characterization of SA is actually based on two main criteria: lethality and intentionality [11]. The lethality corresponds to the probability of irreversible damage according to the SA method [12, 13], and suicidal intent (SI) is defined as the extent to which the patient wished to die at the time of his SA [14]. Recent psychological models have emphasized that suicidal behavior results from the interplay between developmental vulnerabilities, emotional dysregulation, and interpersonal factors [15, 16]. The ideation-to-action framework [17] highlights that the emergence of suicidal ideation and the transition to action may involve distinct mechanisms. Suicidal ideation refers to thoughts of engaging in suicide-related behavior, while SI involves the subjective expectation and desire that the suicidal act will result in death [18]. These conceptual distinctions are particularly relevant in adolescence, where identity, impulsivity, and relational dynamics are in flux [19].

SI is now widely conceptualized as a multidimensional construct, encompassing both objective indicators (such as planning, precautions taken, or lethality) and subjective dimensions (such as desire to die, expectation of death, or ambivalence) [16, 20, 21]. In research on suicidality among adults, SI is frequently evaluated in clinical settings as a marker of suicidal severity, and may serve as a dimension to monitor longitudinally in patients at high risk [22], and strong SI is used as a means of refining our understanding of suicidality and completed suicide [23, 24]. In clinical practice, many guidelines recommend assessing SI [25]. The most widely used assessment scale today is the Suicidal Intent Scale (SIS), which was developed to estimate the degree of SI following a self-aggressive/suicidal act [26, 27]. Other scales are also widely used to assess suicidal behaviors and include items on SI, such as the Columbia Suicide Severity Rating Scale (C-SSRS) [28]. Furthermore, in adult populations, SI has shown prognostic value for long-term suicidal outcomes, emphasizing the importance of better understanding how SI functions in adolescents [29]. While SI has been widely studied and used in adult clinical settings, its evaluation in adolescents is often based on self-report questionnaires, clinician judgment, or general adult-oriented tools that do not consider the specific psychological characteristics of this age group. To date, no widely used and validated instrument exists specifically for assessing SI in adolescents, limiting its clinical application and interpretability [30, 31]. At this age, the reality of SI remains debated [10] when performing a self-directed act, they are no longer in a symbolic elaboration, but rather the result of emotional and psychological difficulties in processing the meaning of death and suicidal behavior, rather than a cognitive inability to imagine death [32–34]. Furthermore, the importance of relational dimension, inherent to any SA in adolescence, may amend perception, expression and assessment of SI [10]. In adolescents, SI appears particularly complex and unstable: early studies already highlighted a frequent mismatch between subjective intent and the medical seriousness of the act. Subsequent studies have further shown that adolescent SI is often marked by greater impulsivity, situational reactivity, and relational motives than in adults, contributing to lower stability and predictability of intent in this age group [30].

Thus, in-depth understanding of the specificities of SI in adolescence, particularly how it is shaped by developmental stage, emotional regulation capacities, and relational dynamics, may help clinicians better interpret suicidal expression, differentiate between ideation and intent, and improve the accuracy and relevance of suicide risk assessment [35]. Its suitable use may enhance predictivity of suicidal reattempt, completed suicide, and reduce morbidity and mortality.

In clinical practice, the evaluation of SI is most often conducted after a SA, where it plays a key role in preventing recurrence. A better understanding of post-attempt SI, particularly in adolescents, may help develop tailored tools for assessing and interpreting suicidal behaviors. In the future, such tools could also inform upstream evaluations to identify high-risk situations before the act occurs. In this review, we also considered the prognostic value of SI, not as a predictor of initial SA, but as a possible indicator of recurrence or long-term risk. To this end, we conducted a systematic review of the literature to examine how SI is expressed and which factors are associated with it in adolescents after a SA.

## Methods

Systematic review of studies of SI in the context of SA in adolescence using the PRISMA (Preferred Reporting Items for Systematic Reviews and Meta-Analyses) protocol [36].

### Study selection

A research group made up of doctors and psychologists specialized in the management of suicidal behavior in adolescence built the study selection protocol, based on a PICO equation combining thesaurus terms and free terms. A detailed description of the successive stages is presented in Additional information.

Eligibility criteria were defined based on the PICO framework, ensuring the inclusion of studies that (I) were published in English or French between 1980 and 2023; (II) included adolescents aged 11–17 years; (III) assessed SI using clinical evaluation, self-report, or hetero-evaluation tools, or qualitatively and (IV) examined SI in the context of SA.

For this review, we specifically focused on studies that assessed SI in the context of a SA in adolescents. We included only studies where SI was explicitly evaluated, through either validated scales, clinical interviews, or detailed descriptions of intent to die. We excluded studies focusing solely on suicidal ideation (including its intensity), suicidal behaviors without explicit assessment of intent, or non-suicidal self-injury (NSSI) where SI was not clearly defined or measured. This criterion was applied to ensure conceptual clarity and comparability across included studies [18]. This review included only studies assessing deliberate self-harm with explicit suicidal intent (DSH-SI).

We included studies published between 1980 and September 2023 to capture the full scope of available research on adolescent SI. This broad range was chosen due to the limited number of eligible studies published after 2005.

The search was carried out in September 2023, using PubMed, EM-premium, Cochrane, Science Direct and CAIRN. Where available, age and titles and abstracts filters were applied.

**Fig. 1:**
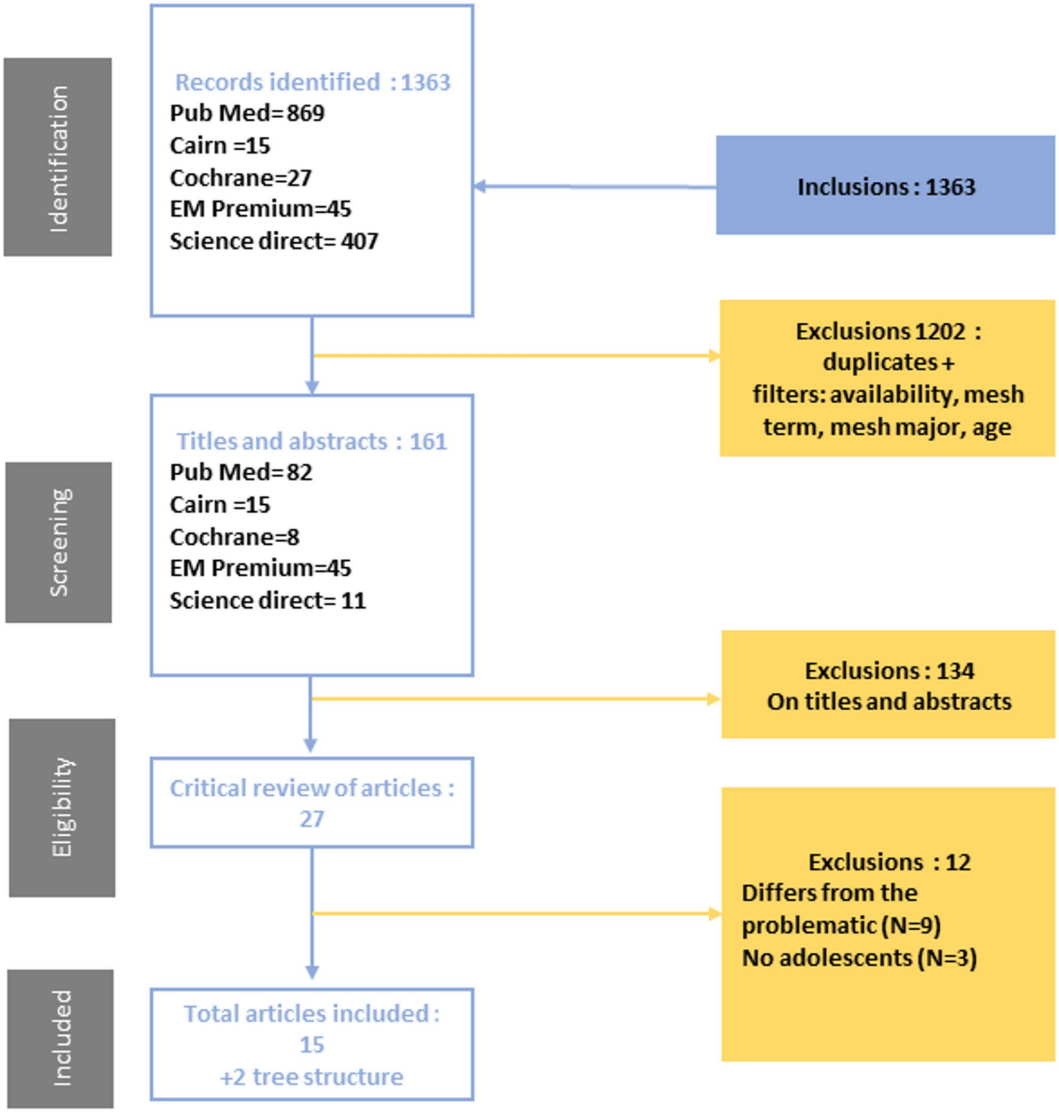
Flow chart of studies screened, identified, or included

Following the first literature review, 1363 studies were identified (Fig. 1). After exclusion of duplicates and application of filters (Additional Information), 161 articles identified as potentially relevant were screened on titles and abstracts by two researchers independently. At this stage, 134 studies were excluded based on their titles and abstracts, due to general non-eligibility (e.g., not focused on suicidal intent, adult samples, unrelated topics) to go fur. The full texts of the remaining 27 articles were retrieved and analyzed, and 12 articles were excluded at this stage: 9 because of their scope outside the theme, and 3 because they did not include adolescents.

Thematic analysis allowed us to group the findings according to four key domains recurrent across the studies: (I) social and familial correlates of SI, (II) clinical and psychiatric correlates, (III) characteristics of the suicidal act and intent, and (IV) the prognostic value of post-attempt SI. This organization aimed to reflect both the determinants and outcomes associated with SI in adolescents after a SA.

### Assessment of study quality

Two reviewers (RS and CR) independently assessed the methodological quality of the included studies using the NIH Quality Assessment Tool for Observational Cohort and Cross-Sectional Studies, Case-control studies and Before After studies [37]. Each study received a global quality rating of “Good”, “Fair”, or “Poor” based on the criteria outlined in the tool. Discrepancies between raters were discussed. Although no formal inter-rater coefficient was calculated, overall agreement between reviewers was high, and disagreements were minimal. A detailed evaluation of each study is provided in the Additional information.

### Study analysis

Selected articles were read then descriptive characteristics were extracted (Table 1 and Additional information). A thematic analysis was then carried out.

**Table 1:** Studies characteristics

Reference	Country	Year	Sample	Population	Methods	Aim of the study	Main results
Brent et al. [38]	USA	1999	*N* = 271	13–19 Male < 16 y.o.: 77.1% (suicide group) vs. 53.6% (control group) Male > 16 y.o.: 87.6% (suicide group) vs. 73.8% (control group)	Observational, case-control	To study age and gender influence on completed suicide	No difference in SI between men and women SI level is higher in adolescents over 16 y.o. (Mann-Whitney U = 1,352.5, *p* = 0.04)
Haw et al. [43]	UK	2015	*N* = 4840	> 12 Male: 37.5% Female: 62.5%	Observational, cross-sectional	To study self-harm features in relation to SI	SIS higher in patients with a psychiatric history (Median [Interquartile Range (IQ)]: 10[5,5–15] vs. 9 [5–13], *p* = 0,02). The number of drugs taken in a drug intoxication is positively correlated with SI measured by the SIS (men: Spearman’s rho = 0.43, *p* < 0.001; women: Spearman’s rho = 0.30, *p* < 0.001). Lethal scenarios have a SIS 6 points higher on average (*p* < 0.001)
Haw and Hawton [ 2]	UK	2008	*N* = 4391	> 15 Male: 35.5% Female: 64.5%	Observational, cross-sectional	To study life problems among self-harmed parents, and their association with SI	Total SI higher for women than for men (Mann-Whitney Z = 6,84, *p* < 0,0001). Women with high suicidal intent have more life problems than those with low suicidal intent (Mann-Whitney Z = 4,095, *p* < 0,0001). Positive correlation between number of problems and SIS score (Spearman = 0,098, 2-tailed *p* < 0,01). Association between high IS and social isolation (men *p* < 0,005, women *p* < 0,0001)
Kumar et al. [13]	India	2006	*N* = 203	> 16 Male (high intent group): 62% Male (low intent group): 44%	Observational, cross-sectional	To explore characteristics associated with a high degree of SI	Positive correlations between high SIS (≥ 11) and depression (49% vs. 13%, *p* < 0,001), schizophrenia (4,8% vs. 0%, *p* = 0,03)
McAuliffe et al. [44]	UK	2007	*N* = 146	> 14 Male: 47% Female: 53%	Observational, cross-sectional	To describe motives and intent of DSH and their association with DSH repetition	Low SIS is positively correlated with escape (*p* < 0.001), and negatively correlated with appeal (*p* < 0.001)
Suominen et al. [29]	Finland	2004	*N* = 224	> 15 Male (suicide group): 53% (*n* = 17) Male (not suicide group): 3% (*n* = 207)	Prospective cohort	To explore risk factors of death by suicide and all causes after attempted suicide	High SIS scores predict death (F = 2,3, df = 221, *p* = 0,02)
Kingsburry [50]	UK	1993	*N* = 50	13–18 Male: 20% Female: 80%	Observational, cross-sectional	To explore SIS’s factors after self-poisoning in adolescents	Low correlation between the two subscales of the SIS, i.e. between declared, subjective intentionality and objective actions when performing SA (α = 0.24, unequal length Spearman-Brown coefficient = 0.39)
Palma-Coca et al. [48]	Mexico	2011	N= 25,056	10–19 “Approximately half were boys”	Observational, cross-sectional	To study links between socioeconomic and health factors and disordered eating behavior	Association between IS and eating disorders (OR : 4,67, IC 95% : 3,01–7,24)
Rotheram-Borus and Trautman [47]	US	1988	*N* = 101	12–17 Female: 100%	Observational, Case-control	To explore SI’s relation with despair and depression	No significant correlation between SI and: depression (Pearson’s correlation *r* = 0,16, not significant) and despair (Pearson’s correlation *r* = 0,17, not significant)
Wang et al. [41]	US	2022	N= 607,838	13–17 N.R.	Retrospective	To compare tendency of calls to the poison control center for adolescents before and during covid-19 pandemic	Increase in drug intoxication with SI during covid period (*p* < 0.001)
Grøholt et al. [39]	Norway	2000	*N* = 92	13–19 Male: 10% Female: 90%	Observational, cross-sectional	To compare risk factors, SA’s circumstances and relationships among adolescents with high and low intent after a SH	Low SIS is associated with emotional disruptivity and impulsivity (standard deviation) : 7,2(4,6) when impulsive SA vs. 14,3(4,9) when premeditated SA, *p* < 0,001)
Zhang et al. [45]	China	2017	*N* = 791	15–54 Male: 37% Female: 63%	Observational, cross-sectional	To study relationships between the level of life satisfaction and the degree of suicide intent among people who attempted suicide	Having more years of education is related to a lower level of SIS (*p* = 0.011). Peasants score higher on SIS than the non-peasants (*p* = 0.043). Those living alone score higher on SIS than those not living along (*p* = 0.021). Those with suicide attempt in the family score higher on SIS than those who do not have such a family member (*p* = 0.035)
Sapyta et al. [51]	US	2012	*N* = 180	12–19 Male: 49% Female: 51%	Prospective cohort	To investigate the predictive value of SI and lethality of SA on the risk of subsequent attempts, and the links between intent and lethality	The intent of the most recent attempt was not predictive of the time until future suicide attempts (*p* = 0.82). Maximum intent of prior attempts was found to be predictive of subsequent suicide attempts (b = 0.749, se = 0.222, HR = 2.12, *p* < 0.001). Maximum values of intent also were predictive of future attempts even after considering the number of prior attempts (b = 0.655, se = 0.237, HR = 1.93, *p* < 0.01)
Mars et al. [40]	UK	2014	*N* = 4799	16 Female who had self-harmed with suicidal intent: 81.2%	Prospective cohort with retrospective subgroup analysis	To study the differences between DSH with and without SI	Participants who had self-harmed with suicidal intent at some point during their lifetime were more likely than those who self-harmed without suicidal intent to have taken an overdose on the most recent occasion (28% vs. 5%, risk difference 0.23 [95% CI 0.17, 0.28])
Parellada et al. [46]	Spain	2008	*N* = 104	12–17 Male: 22.1% Female: 77.9%	Retrospective	To compare SAs between adolescent and adult population	The difference in SI was statistically significant between adults and adolescents, with more adults having high intent (χ2 = 24.63, df = 2, P b 0.001).
Guertin et al. [49]	US	2001	*N* = 95	12–18 Male: 16% Female: 84%	Retrospective	To compare adolescents with and without DSH history after a SA	No significative difference on SIS scores between groups with and without SH history
Freedenthal [31]	US	2008	N= 29,264	> 16 Male: 38.4% Female: 61.6% (among 98 unique studies reporting gender composition)	Systematic review and meta-analysis	To summarize existing data on SIS	The mean SIS score increased in concert with age, and statistically significant differences existed between adolescents and the remaining older age groups

## Results

Seventeen studies were included in this review, the characteristics of which are detailed in Table 1. Six studies were conducted in the United States, 8 in Europe, 2 in Asia, and 1 in Mexico. 75% of the studies were carried out before 2013. Among the studies, half are cross-sectional studies, two retrospective cohorts, two case-control studies and one before-after study.

Among the 17 studies included, the majority relied on clinical samples (hospitalized or outpatient adolescents), while a minority was based on school or community populations. Sample characteristics, including setting and population details, are summarized in Table 1 and in the Additional Information document.

The method used to assess SI is detailed in Table 1. Among the 17 included studies, 11 used the Beck SIS, 2 used the Pierce Suicide Intent Scale, 2 relied on self-reported SI, 2 employed clinician judgment or expert choice, and 1 used the SIRS.

The results are organized around four main themes describing the (I) Social and familial correlates of SI; (II) Clinical and psychiatric correlates of SI; (III) Characteristics of the SA and intent; (IV) Prognostic value of post-attempt SI, that are associated with SI in adolescents after a SA.

### Social and Familial correlates of SI

The link between social and environmental profile and SI in the adolescent population was explored in eleven of the included articles. Of these, five were specific to the adolescent population. Low income, modest social status, low maternal education, and low adolescent IQ score were associated with DSH-SI among participants [13, 38–46].

About age, three studies highlighted significant differences concerning the level of SI between different age groups: two studies found lower level of SI for adolescents under 16 than for those over 16/adults [31, 38] and one study found higher level in adults than in adolescents (for females only) [46]. However, one study discovered no differences between those age groups [39].

Concerning gender, four studies spotted no differences in SI between male and female in different context [38, 39, 44, 45]. In three other studies, significant differences between gender were pinpointed, all of them finding higher SI in men than in women [13, 42, 43].

Concerning ethnicity, no significant differences were highlighted between “intent to die” and “other intent” groups [39].

About socio-economic status, two studies encountered no significant associations between the level of education, the occupation or the location and the level of SI [13, 39]. However, three studies found some associations with the environment. For example, higher SI was significantly associated with social isolation [42, 45], low income or modest social status [40] or inversely associated with school education level or low adolescent IQ [40, 45].

One study conducted during the Covid-19 pandemic reported an increase in intentional self-poisonings associated with SI. Although this is an isolated finding, it suggests that environmental stressors such as the pandemic may influence the modalities and expression of SI [41].

### Clinical and psychiatric correlates of SI

Ten articles, five of which included only adolescent subjects, studied the correlations between SI and clinical profiles. Seven of them highlighted significant differences concerning the association between SI and internalized disorders and three of them reported no differences. Finally, six of them also found differences concerning externalized disorders [13, 39, 40, 42–45, 47–49].

### Internalized disorders

Concerning depression and hopelessness, one study spotted no significant correlation between SI, depression and hopelessness in girls [47] and two studies related the opposite: hopelessness was significantly correlated with the high SI group and the “intent to die” group showed more depression in the first one [39]; psychiatric disorders were significantly and globally associated with SI, and more specifically with depression and schizophrenia in the second one [13].

Concerning eating disorders (ED) and low body satisfaction, Haw and Hawton (2008) and Palma-Coca et al. (2011) discovered a significant association between SI and ED [42, 48]. Zhang et al. (2017) showed a significant and positive association between SI and low body satisfaction [45].

Four studies found association between SI and somatic illness or traumatic and family history. For Zhang et al. (2017), SI was significantly associated with the existence of somatic illness in the participants [45]. For Mars et al. (2014), DSH with SI was significantly associated with traumatic and family histories and childhood sexual abuse was a risk factor [40]. In the study of Kumar et al. (2006) and Haw and Hawton (2008), SI was associated with the number of stressful life events or life problems [13, 42].

Finally, in four studies, SI was significantly associated with psychiatric disorders or acute psychiatric care [13, 42, 43, 45].

### Externalized disorders

About deliberate self-harm (DSH), two studies assessed histories of self-harm without specifying intent, but highlighted no significant association with SI as assessed at the time of the SA [44, 49]. However, four studies found that SI was significantly associated with a history of DSH [40, 42, 43, 45].

Concerning substance use (alcohol, tobacco and drug poisoning), five studies showed significant associations with SI. Grøholt et al. (2000) found that the “other-intent” group had more substance use disorders than the “intent to die group” [39]. Haw and Hawton (2008), Kumar et al. (2006), Mars et al. (2014), and Haw et al. (2015) found that alcohol use disorders were inversely and significantly associated with SI [13, 40, 42, 43]. However, for Haw et al. (2015), the association with alcohol consumption varied according to the time of intake (negative if consumption preceded the suicidal act, positive if consumption was concomitant with the suicidal act). In this study, DSH with SI was also significantly associated with tobacco use, and cannabis use was a risk factor similarly associated [40]. When the method of SA was drug self-poisoning, SI was higher when the self-poisoning was polymedicated, with a weakly positive correlation between number of tablets and SI [43].

Finally, Grøholt et al. (2000) showed that the “other intent group” had greater school absenteeism than the “intent to die group” [39].

### Characteristics of the SA and intent: motives, precipitating factors, methods

The various components of the suicidal act and their link with SI were explored in three articles (two of them studied the adolescent population only) [39, 44, 50].

 Interpersonal and impulsivity-related motivation were inversely and significantly associated with SI, as highlighted by Grøholt et al. (2000) [39].

A small correlation between self-reported intentionality and the objective actions surrounding the SA measured was found in Kingsburry et al. (1993) [50].

Finally in McAuliffe’s study (2007), SI was significantly positively associated with DSH motives associated with escape (*Unbearable thoughts*,* Unbearable situation*,* I wanted to die*,* I wanted to make things easier for others*) and significantly negatively related with motives associated with interruption (*I lost control and I don’t know why I did it*,* I wanted to get away for a while*,* I wanted others to know how desperate I felt*,* I wanted to sleep for a while*) and appeal (*I wanted to show love*,* I wanted to get help*,* I wanted to know if someone cared*,* I wanted to persuade someone to change mind*) [44].

### Prognostic value of post-attempt SI: lethality, SA risk and death by suicide

Finally, four articles – two of them were specific to the adolescent population – explored the prognostic dimension of SI [29, 39, 43, 51].

Sapyta et al. (2012) spotted that SI associated with the most recent episode was not predictive of future suicidal behavior, but the maximum level of intent across all prior attempts was significantly associated with future risk, even after adjusting for the number of previous attempts and methods used. Four studies explored the lethality. They found a correlation between higher SI scores and high-lethal-risk SA [29, 39, 43]. However, for Sapyta et al. (2012), with regard to the link between SI and lethality, the authors didn’t find any correlation in the under-20 age group, unlike in adults (where a positive correlation exists) [51].

## Discussion

The findings of this systematic review highlight several key aspects of SI in adolescents. SI appears to increase with age in intensity, with higher levels observed in adolescents over 16 years old compared to younger adolescents, and even higher levels in adults. The influence of gender on SI remains unclear, as studies provide conflicting results, leading to the hypothesis that there is no clear significant difference between males and females. SI is more strongly linked to internalized psychiatric disorders, such as depression and eating disorders, while it appears to be inversely associated with externalized disorders. Although SI is often associated with internalizing psychiatric disorders, no diagnosis has demonstrated consistent predictive power for SA in adolescents [52]. Unlike in adults it is not evident that high SI would be closely associated with feelings of despair. Additionally, impulsivity is correlated with lower SI at the time of a SA. The association between SI and SA methods remains inconsistent across studies, and while SI is generally linked to more severe SA, there is no clear evidence of a direct association between intentionality and lethality of the attempt, contrary to findings in adults. Importantly, the highest reported level of SI across multiple SA episodes appears to be a significant prognostic factor for future SA and long-term risk of death by suicide or other causes. In the adult population, SI is used as a tool for assessing SAs. SI is a predictive factor for the repetition of SAs and for completed suicide [31, 53–57]. Although other methods of assessing SI exist, for the past 40 years it has been assessed almost exclusively by the SIS, which has thus become the Gold Standard [27, 31, 58]. In particular, it showed non-inferiority compared with other scales for suicidal risk assessment [59, 60].

These findings collectively highlight the limitations of existing approaches to assessing SI in adolescents. Most of the included studies used the Beck SIS, which, as a global score, is not fully appropriate for adolescents, and none employed the three-factor adolescent-adapted version proposed by Spirito et al. (1996) [30]. The observed variability in evaluation methods, including the use of self or hetero-reported assessments, further reflects the lack of consensus on how to capture SI in this age group. Combined with the underrepresentation of relational and contextual factors, these observations underscore the need for tools specifically adapted to the developmental and psychosocial specificities of adolescents.

However, the adolescent’s relationship with death is not the same as that of the adult, and SI should not be approached or assessed in the same way. In fact, adolescence is a period of time when the idea of non-reversibility of death emerges, but is not yet fully consolidated. SAs therefore sometimes take place without any real awareness of the irreversibility of a potential death [35]. The suicidal act would then be situated in *doing* without having the possibility of *thinking about* it. What’s more, frequently in adolescence, the act of suicide is not exclusively part of an escape, but also has a message value in often complex personal and environmental situations [61]. The inclusion of the interpersonal component, particularly with parents, in the evaluation of the adolescent’s SI would allow for a more appropriate assessment [10].

Our review shows that SI appears to evolve with age, progressively increasing from early to late adolescence, as neurocognitive capacities mature and the mentalization of death becomes more stable [31, 38, 46, 62]. It therefore seems necessary to specify the age range in which SI measurement is relevant, possibly more so in the older age groups (15–18 years), which are also those most likely to attempt suicide [63–65]. This is consistent with the instability of the mentalization of non-reversibility of death at this age. In the same way, impulsivity is associated with low SI, arguing for more *doing* and less *thinking about* in adolescenthood SA. In this idea, SI may be understood as a work in progress in adolescence, in parallel with the neurocognitive development [66, 67]. SI would then fluctuate, gradually increasing with age in adolescents with suicidal ideas just as mentalization of death becomes more refined with age [62], with sharp rises around SA.

The links between SI and psychiatric disorders seem less clear in adolescence than in adulthood, as shown by the discordant results of the various studies included. This result may be explained by the importance of the environment in the genesis, maintenance and resolution of adolescent psychiatric disorders. In particular, the impact of familial and relational difficulties on psychic disorders, which are more prevalent at this age, could explain the lack of concordance of results with adult data, especially associations of SI with depression or despair [53, 68–72].

In adolescents who have attempted suicide, the clinical variability in SI may reflect different internal processes. Internalizing disorders are frequently associated with high and persistent levels of SI. In contrast, externalizing traits may influence the transition to action through impulsivity or impaired mentalization [52, 73]. These findings are consistent with theoretical models distinguishing suicidal ideators from attempters, which emphasize the role of acquired capability and behavioral dysregulation in facilitating the shift to action [17]. This may also explain why the quality or intensity of SI is not always correlated with the lethality or planning of the act. Additionally, the retrospective nature of intent assessment raises concerns, particularly in adolescents who may experience dissociative states during the act, limiting the reliability of self-reported SI [34].

The main finding of our study is the prognostic value of SI in relation to long-term risk of death in adolescents who have attempted suicide. This finding highlights the importance of assessing SI not only during the most recent episode, but also across the individual’s full history of suicidal behavior [51]. The peak intensity of intent may serve as a more stable marker of long-term suicide risk, and could be integrated into clinical risk assessments and follow-up planning. In adults, a high SI is predictive of death by suicide, and the results of our review show that the same may be true for adolescents. Assessment of SI would then enable us to adjust the level of care for adolescents who have presented a high level of SI during a SA. In view of this finding, it seems essential to include in routine the assessment of SI in adolescence to modulate the medium-term prognosis, and the big challenge is to build adolescents’ specific tools for this job. Although some included studies report associations between SI and recurrence or mortality, this does not reflect a focus on prediction of first attempts. Rather, it highlights the possible prognostic value of post-attempt SI in adolescents, which may inform follow-up and prevention strategies.

Indeed, no study has specifically measured SI in adolescents since 2005 [27, 50]. The only tool adapted to the adolescent population was developed by Spirito et al. (1996) and adapted from the adult SIS. The authors showed that in adolescents, interpretation of the SIS would be more efficient using a three-factor version (“Expected Outcome”, “Planning Activities” and “Isolation Behaviors”). Although Spirito’s SIS is a promising scale, it has never been used in large-scale studies [30, 74], and it is not used in clinical practice. We then need to develop and test assessment tools staking account to the particularities of the different adolescent population (i.e. young and old adolescents) and the importance of environmental, familial and relational influence on SAs and SI [10].

It’s true that the relational component of SA in adolescence, particularly with parents, is a major element, and should be for SI as well [75, 76]. It is also indispensable to consider the parents’ opinion in the assessment of a suicidal crisis [77], as it could have an impact on the prognostic factors of suicidal crisis [78]. Such as studying the adolescent’s broader environment to understand the meaning associated with SA [79]. Parental intervention has an impact on the evolution of the suicidal crisis [80], whether through the evolution of their representations and attitudes following the SA that may lead to positive or negative evolution of suicidal behaviors (control vs. caregiving) [81]. Considering their participation in the assessment of SI after a SA could help integrate them as co-therapists with the aim of achieving a favorable outcome to the suicidal crisis [82, 83].

The lack of recent studies on SI in adolescents leaves several key gaps. Longitudinal research is needed to understand how SI evolves with age and interacts with impulsivity, mental health disorders, social factors, suicidal motivations. Current assessment tools, mostly adapted from adults, are not validated for adolescents, highlighting the need for specific, standardized measures. Further studies should also examine SI’s predictive value in suicide risk, particularly its role alongside known risk factors like previous attempts and hopelessness. Qualitative research could help clarify the contextual factors influencing SI, improving prevention and intervention strategies. Qualitative studies exploring the relational and familial dimensions of SI in adolescence could also provide valuable foundations for the development of tailored, developmentally sensitive assessment tools [84].

The findings of this review highlight the importance of incorporating a developmentally informed assessment of SI in adolescents after a SA. Beyond improving the understanding of suicidal processes, this approach may help clinicians refine the evaluation of recurrence risk and tailor follow-up strategies. Given the emotional, cognitive, and relational specificities of adolescence, there is a need to develop or adapt assessment tools that go beyond adult-based measures. Moreover, including parents or caregivers in the post-attempt assessment may offer complementary insights and promote therapeutic alliance. These perspectives can guide clinical practices and prevention strategies in both mental health and other contexts.

The limitations of our study are mainly due to the lack of literature on SI in adolescence. More than a third of the studies included in our review were partly based on an adult population, without it always being possible to individualize the adolescent population. We also note the lack of recent data: only three studies have been published in the last ten years. Finally, the disparity in the assessment of SI and the heterogeneity of the results make it impossible to give a clear ruling on the different aspects studied.

## Conclusion

In this systematic review, we explored how SI is expressed and assessed in adolescents after a suicide attempt. The results highlight the heterogeneity of existing approaches, the absence of validated age-specific tools, and the underrepresentation of relational and contextual dimensions in the assessment process. SI seems to be higher in intensity in suicidal older adolescents, with no clear gender difference, and associated with internalized disorders. The maximum level of SI after SAs appears to be one of the prognostic factors for longitudinal death by suicide and other causes. While none of the included studies demonstrated a strong predictive value of SI for new suicide attempts, some findings suggest that the maximum level of SI across past attempts may have prognostic relevance for long-term suicide-related outcomes, including mortality.

These findings emphasize that, although current measures fall short of supporting SI as a reliable predictive indicator in adolescents, its predictive value might emerge if assessed with developmentally appropriate, multidimensional tools that account for the emotional, cognitive, and interpersonal characteristics of adolescence.

Future research should aim to develop and validate such tools, potentially improving suicide risk assessment and the targeting of post-attempt interventions in this population.

## Supplementary Information

Below is the link to the electronic supplementary material.

Supplementary material 1.

## Data Availability

No datasets were generated or analysed during the current study.

## References

[1] Ward JL, Azzopardi PS, Francis KL, Santelli JS, Skirbekk V, Sawyer SM, et al. Global, regional, and National mortality among young people aged 10–24 years, 1950–2019: a systematic analysis for the global burden of disease study 2019. Lancet. 2021;398(10311):1593–618. 34755628 10.1016/S0140-6736(21)01546-4 PMC8576274

[2] Galynker I, Yaseen ZS, Cohen A, Benhamou O, Hawes M, Briggs J. Prediction of suicidal behavior in high risk psychiatric patients using an assessment of acute suicidal state: the suicide crisis inventory: Galynker et al. Depress Anxiety. 2017;34(2):147–58. 27712028 10.1002/da.22559

[3] Brener ND, Krug EG, Simon TR. Trends in suicide ideation and suicidal behavior among high school students in the united States, 1991–1997. Suicide Life Threat Behav. 2000;30(4):304–12. 11210056

[4] Nelson HD, Denneson LM, Low AR, Bauer BW, O’Neil M, Kansagara D, et al. Suicide risk assessment and prevention: A systematic review focusing on veterans. PS. 2017;68(10):1003–15. 10.1176/appi.ps.201600384 28617209

[5] Large M, Kaneson M, Myles N, Myles H, Gunaratne P, Ryan C. Meta-Analysis of Longitudinal Cohort Studies of Suicide Risk Assessment among Psychiatric Patients: Heterogeneity in Results and Lack of Improvement over Time. DeLuca V, editor. PLoS ONE. 2016;11(6):e0156322. 10.1371/journal.pone.0156322 PMC4902221 27285387

[6] Cousien A, Acquaviva E, Kernéis S, Yazdanpanah Y, Delorme R. Temporal trends in suicide attempts among children in the decade before and during the COVID-19 pandemic in Paris, France. JAMA Netw Open. 2021;4(10):e2128611. 34618041 10.1001/jamanetworkopen.2021.28611 PMC8498848

[7] Mourouyave M, Bottemanne H, Bonny G, Angoulvant F, Cohen J, Ouss L. Association between suicide behaviours in children and adolescents and the COVID-19 lockdown in Paris, france: a retrospective observational study. Arch Dis Child. 2021;106(11):e42–42. 33355154 10.1136/archdischild-2020-320628 PMC8380898

[8] Borges G, Nock MK, Haro Abad JM, Hwang I, Sampson NA, Alonso J, et al. Twelve-Month prevalence of and risk factors for suicide attempts in the world health organization world mental health surveys. J Clin Psychiatry. 2010;71(12):1617–28. 20816034 10.4088/JCP.08m04967blu PMC3000886

[9] Cornaggia C, Beghi. Rosenbaum, Cerri. Risk factors for fatal and nonfatal repetition of suicide attempts: a literature review. NDT. 2013;1725. 10.2147/NDT.S40213 PMC3825699 24235836

[10] Sibut R, Lachal J. The interpersonal component of suicidal intent in the assessment of adolescent suicidal crisis. Eur Arch Psychiatry Clin Neurosci [Internet]. 2024 Sep [cited 2024 Sep 23]; Available from: https://link.springer.com/10.1007/s00406-024-01907-8 10.1007/s00406-024-01907-8 39307885

[11] Misson H, Mathieu F, Jollant F, Yon L, Guillaume S, Parmentier C, et al. Factor analyses of the suicidal intent scale (SIS) and the Risk-Rescue rating scale (RRRS): toward the identification of homogeneous subgroups of suicidal behaviors. J Affect Disord. 2010;121(1–2):80–7. 19524302 10.1016/j.jad.2009.05.012

[12] Weisman AD. Risk-Rescue rating in suicide assessment. Arch Gen Psychiatry. 1972;26(6):553. 5027119 10.1001/archpsyc.1972.01750240065010

[13] Kumar C, Mohan R, Ranjith G, Chandrasekaran R. Characteristics of high intent suicide attempters admitted to a general hospital. J Affect Disord. 2006;91(1):77–81. 16443283 10.1016/j.jad.2005.12.028

[14] Hawton K, James A. Suicide and deliberate self harm in young people. BMJ. 2005;330(7496):891–4. 15831877 10.1136/bmj.330.7496.891 PMC556165

[15] Barzilay S, Apter A. Psychological models of suicide. Archives Suicide Res. 2014;18(4):295–312. 10.1080/13811118.2013.824825 24568371

[16] Klonsky ED, May AM, Saffer BY, Suicide. Suicide Attempts, and suicidal ideation. Annu Rev Clin Psychol. 2016;12(1):307–30. 26772209 10.1146/annurev-clinpsy-021815-093204

[17] Klonsky ED, May AM. Differentiating suicide attempters from suicide ideators: A critical frontier for suicidology research. Suicide Life Threat Behav. 2014;44(1):1–5. 24313594 10.1111/sltb.12068

[18] Silverman MM, Berman AL, Sanddal ND, O’Carroll PW, Joiner TE. Rebuilding the tower of babel: A revised nomenclature for the study of suicide and suicidal behaviors part 1: Background, Rationale, and methodology. Suicide Life Threat Behav. 2007;37(3):248–63. 17579538 10.1521/suli.2007.37.3.248

[19] Gatta M, Raffagnato A, Angelico C, Benini E, Medda E, Fasolato R, et al. Externalising Behaviours, Impulsivity, Alexithymia, and emotional dysregulation in adolescents’ suicidality. Clin Neuropsychiatry. 2023;20(1):17–28. 36936619 10.36131/cnfioritieditore20230103 PMC10016105

[20] Nock MK, Borges G, Bromet EJ, Cha CB, Kessler RC, Lee S. Suicide and suicidal behavior. Epidemiol Rev. 2008;30(1):133–54. 18653727 10.1093/epirev/mxn002 PMC2576496

[21] Szücs A, Perry-Falconi MA, O’Brien EJ, Keilp JG, Bridge JA, Maier AB, et al. Objective and subjective suicidal intent are differentially associated with attempt lethality based on age of onset of suicidal behavior. Sci Rep. 2025;15(1):5621. 39955394 10.1038/s41598-025-89844-x PMC11829946

[22] Hasley JP, Ghosh B, Huggins J, Bell MR, Adler LE, Shroyer ALW. A review of suicidal intent within the existing suicide literature. Suicide Life Threat Behav. 2008;38(5):576–91. 19014309 10.1521/suli.2008.38.5.576

[23] Horesh N, Levi Y, Apter A. Medically serious versus non-serious suicide attempts: relationships of lethality and intent to clinical and interpersonal characteristics. J Affect Disord. 2012;136(3):286–93. 22197510 10.1016/j.jad.2011.11.035

[24] Levi-Belz Y, Gvion Y, Apter A. The serious suicide attempts approach for Understanding suicide: review of the psychological evidence. Omega (Westport). 2022;86(2):591–608. 33327864 10.1177/0030222820981235

[25] Bernert RA, Hom MA, Roberts LW. A review of multidisciplinary clinical practice guidelines in suicide prevention: toward an emerging standard in suicide risk assessment and Management, training and practice. Acad Psychiatry. 2014;38(5):585–92. 25142247 10.1007/s40596-014-0180-1 PMC4283785

[26] Beck AT. Classification of suicidal behaviors: II. Dimensions of suicidal intent. Arch Gen Psychiatry. 1976;33(7):835. 942287 10.1001/archpsyc.1976.01770070065006

[27] Beck RW, Morris JB, Beck AT. Cross-Validation of the suicidal intent scale. Psychol Rep. 1974;34(2):445–6. 4820501 10.2466/pr0.1974.34.2.445

[28] Posner K, Subramany R, Amira L, John Mann J. From Uniform Definitions to Prediction of Risk: The Columbia Suicide Severity Rating Scale Approach to Suicide Risk Assessment. In: Cannon KE, Hudzik TJ, editors. Suicide: Phenomenology and Neurobiology [Internet]. Cham: Springer International Publishing; 2014 [cited 2025 Oct 3]. pp. 59–84. Available from: https://link.springer.com/10.1007/978-3-319-09964-4_4

[29] Suominen K, Isometsä E, Ostamo A, Lönnqvist J. Level of suicidal intent predicts overall mortality and suicide after attempted suicide: a 12-year follow-up study. BMC Psychiatry. 2004;4(1):11. 15099401 10.1186/1471-244X-4-11 PMC415554

[30] Spirito A, Sterling CM, Donaldson DL, Arrigan ME. Factor analysis of the suicide intent scale with adolescent suicide attempters. J Pers Assess. 1996;67(1):90–101. 8683428 10.1207/s15327752jpa6701_7

[31] Freedenthal S. Assessing the wish to die: A 30-Year review of the suicide intent scale. Archives Suicide Res. 2008;12(4):277–98. 10.1080/13811110802324698 18828032

[32] Grandclerc S, De Labrouhe D, Spodenkiewicz M, Lachal J, Moro MR. Relations between Nonsuicidal Self-Injury and Suicidal Behavior in Adolescence: A Systematic Review. Botbol M, editor. PLoS ONE. 2016;11(4):e0153760. 10.1371/journal.pone.0153760 PMC4835048 27089157

[33] Williams R, Fiorentino F, Lingiardi V, Moselli M, Sharp C, Tanzilli A. The assessment of pathways towards suicide in adolescent patients: A PDM-2‐oriented approach. Psychol Psychother. 2025;98(2):289–307. 38742777 10.1111/papt.12529

[34] Casini MP, Moselli M, Wisniewski A, Williams R. The role of suicidal motivations in adolescence: implications for the psychotherapeutic treatment of suicidal risk. Res Psychother. 2024;27(3):833. 39628385 10.4081/ripppo.2024.833 PMC11822347

[35] Grandclerc S, Spiers S, Spodenkiewicz M, Moro MR, Lachal J. The quest for meaning around Self-Injurious and suicidal acts: A qualitative study among adolescent girls. Front Psychiatry. 2019;10:190. 31024356 10.3389/fpsyt.2019.00190 PMC6461024

[36] Sohrabi C, Franchi T, Mathew G, Kerwan A, Nicola M, Griffin M, et al. PRISMA 2020 statement: what’s new and the importance of reporting guidelines. Int J Surg. 2021;88:105918. 33789825 10.1016/j.ijsu.2021.105918

[37] Ma LL, Wang YY, Yang ZH, Huang D, Weng H, Zeng XT. Methodological quality (risk of bias) assessment tools for primary and secondary medical studies: what are they and which is better? Military Med Res. 2020;7(1):7. 10.1186/s40779-020-00238-8 PMC7049186 32111253

[38] Brent DA, Baugher M, Bridge J, Chen T, Chiappetta L. Age- and Sex-Related risk factors for adolescent suicide. J Am Acad Child Adolesc Psychiatry. 1999;38(12):1497–505. 10596249 10.1097/00004583-199912000-00010

[39] Grøholt B, Ekeberg Ø, Haldorsen T. Adolescents hospitalised with deliberate self-harm: the significance of an intention to die. Eur Child Adolesc Psychiatry. 2000;9(4):244–54. 11202099 10.1007/s007870070027

[40] Mars B, Heron J, Crane C, Hawton K, Kidger J, Lewis G, et al. Differences in risk factors for self-harm with and without suicidal intent: findings from the ALSPAC cohort. J Affect Disord. 2014;168:407–14. 25108277 10.1016/j.jad.2014.07.009 PMC4160300

[41] Wang GS, Leonard J, Cornell A, Hoyte C. Adolescent US poison center exposure calls during the COVID-19 pandemic. J Adolesc Health. 2022;71(6):764–7. 36088226 10.1016/j.jadohealth.2022.07.014 PMC9451938

[42] Haw C, Hawton K. Life problems and deliberate self-harm: associations with gender, age, suicidal intent and psychiatric and personality disorder. J Affect Disord. 2008;109(1–2):139–48. 18221789 10.1016/j.jad.2007.12.224

[43] Haw C, Casey D, Holmes J, Hawton K. Suicidal intent and method of Self-Harm: A Large-scale study of Self-Harm patients presenting to a general hospital. Suicide Life Threat Behav. 2015;45(6):732–46. 25916308 10.1111/sltb.12168

[44] McAuliffe C, Arensman E, Keeley HS, Corcoran P, Fitzgerald AP. Motives and suicide intent underlying hospital treated deliberate Self-Harm and their association with repetition. Suicide Life-Threatening Behav. 2007;37(4):397–408. 10.1521/suli.2007.37.4.397 17896880

[45] Zhang J, Liu Y, Sun L. Life satisfaction and degree of suicide intent: A test of the strain theory of suicide. Compr Psychiatr. 2017;74:1–8. 10.1016/j.comppsych.2016.12.002 28040550

[46] Parellada M, Saiz P, Moreno D, Vidal J, Llorente C, Álvarez M, et al. Is attempted suicide different in adolescent and adults? Psychiatry Res. 2008;157(1–3):131–7. 17888518 10.1016/j.psychres.2007.02.012

[47] Rotheram-Borus MJ, Trautman PD. Hopelessness, Depression, and suicidal intent among adolescent suicide attempters. J Am Acad Child Adolesc Psychiatry. 1988;27(6):700–4. 3198555 10.1097/00004583-198811000-00006

[48] Palma-Coca O, Hernández-Serrato MI, Villalobos-Hernández A, Unikel-Santoncini C, Olaiz-Fernández G, Bojorquez-Chapela I. Association of socioeconomic Status, problem Behaviors, and disordered eating in Mexican adolescents: results of the Mexican National health and nutrition survey 2006. J Adolesc Health. 2011;49(4):400–6. 21939871 10.1016/j.jadohealth.2011.01.019

[49] Guertin T, Lloyd-Richardson E, Spirito A, Donaldson D, Boergers J. Self-Mutilative behavior in adolescents who attempt suicide by overdose. J Am Acad Child Adolesc Psychiatry. 2001;40(9):1062–9. 11556630 10.1097/00004583-200109000-00015

[50] Kingsbury SJ. Clinical components of suicidal intent in adolescent overdose. J Am Acad Child Adolesc Psychiatry. 1993;32(3):518–20. 8496114 10.1097/00004583-199305000-00005

[51] Sapyta J, Goldston DB, Erkanli A, Daniel SS, Heilbron N, Mayfield A, et al. Evaluating the predictive validity of suicidal intent and medical lethality in youth. J Consult Clin Psychol. 2012;80(2):222–31. 22250854 10.1037/a0026870 PMC3314156

[52] Bilsen J. Suicide and youth: risk factors. Front Psychiatry. 2018;9:540. 30425663 10.3389/fpsyt.2018.00540 PMC6218408

[53] Harriss L, Hawton K, Zahl D. Value of measuring suicidal intent in the assessment of people attending hospital following self-poisoning or self-injury. Br J Psychiatry. 2005;186(1):60–6. 15630125 10.1192/bjp.186.1.60

[54] Hawton K, Harriss L. Deliberate self-harm in young people: characteristics and subsequent mortality in a 20-year cohort of patients presenting to hospital. J Clin Psychiatry. 2007;68(10):1574–83. 17960975

[55] Niméus A, Én M, Träskman-Bendz L. High suicidal intent scores indicate future suicide. Archives Suicide Res. 2002;6(3):211–9.

[56] Hjelmeland H. Verbally expressed intentions of parasuicide: II. Prediction of fatal and nonfatal repetition. Crisis. 1996;17(1):10–4. 8768401 10.1027/0227-5910.17.1.10

[57] Stefansson J, Nordström P, Jokinen J. Suicide intent scale in the prediction of suicide. J Affect Disord. 2012;136(1–2):167–71. 21144592 10.1016/j.jad.2010.11.016

[58] Sisask M, Kõlves K, Värnik A. Severity of attempted suicide as measured by the Pierce suicidal intent scale. Crisis. 2009;30(3):136–43. 19767269 10.1027/0227-5910.30.3.136

[59] Lindh ÅU, Beckman K, Carlborg A, Waern M, Salander Renberg E, Dahlin M, et al. Predicting suicide: A comparison between clinical suicide risk assessment and the suicide intent scale. J Affect Disord. 2020;263:445–9. 31969276 10.1016/j.jad.2019.11.131

[60] Runeson B, Odeberg J, Pettersson A, Edbom T, Jildevik Adamsson I, Waern M. Instruments for the assessment of suicide risk: A systematic review evaluating the certainty of the evidence. Abe T, editor. PLoS ONE. 2019;12(7):e0180292. 10.1371/journal.pone.0180292 PMC5517300 28723978

[61] Janiri D, Doucet GE, Pompili M, Sani G, Luna B, Brent DA, et al. Risk and protective factors for childhood suicidality: a US population-based study. Lancet Psychiatry. 2020;7(4):317–26. 32171431 10.1016/S2215-0366(20)30049-3 PMC7456815

[62] Gudas LJ. Concepts of Death and Loss in Childhood and Adolescence. In: Saylor CF,Children and Disasters [Internet]., Boston MA. Springer US; 1993 [cited 2024 Oct 29]. pp. 67–84. (Roberts MC, Peterson L, editors. Issues in Clinical Child Psychology). Available from: http://link.springer.com/10.1007/978-1-4757-4766-9_5

[63] Glenn CR, Lanzillo EC, Esposito EC, Santee AC, Nock MK, Auerbach RP. Examining the course of suicidal and nonsuicidal Self-Injurious thoughts and behaviors in outpatient and inpatient adolescents. J Abnorm Child Psychol. 2017;45(5):971–83. 27761783 10.1007/s10802-016-0214-0 PMC5397367

[64] Spiller HA, Ackerman JP, Smith GA, Kistamgari S, Funk AR, McDermott MR, et al. Suicide attempts by self-poisoning in the united States among 10–25 year olds from 2000 to 2018: substances used, Temporal changes and demographics. Clin Toxicol. 2020;58(7):676–87. 10.1080/15563650.2019.1665182 31587583

[65] Spiller HA, Ackerman JP, Spiller NE, Casavant MJ. Sex- and Age-specific increases in suicide attempts by Self-Poisoning in the united States among youth and young adults from 2000 to 2018. J Pediatr. 2019;210:201–8. 31054768 10.1016/j.jpeds.2019.02.045

[66] Ortuño-Sierra J, Aritio-Solana R, del Casal ADG, Fonseca-Pedrero E. Neurocognitive functioning in adolescents at risk for suicidal behaviors. Archives Suicide Res. 2021;25(3):657–71. 10.1080/13811118.2020.1746938 32264769

[67] Luna B, Tervo-Clemmens B, Calabro FJ. Considerations when characterizing adolescent neurocognitive development. Biol Psychiatry. 2021;89(2):96–8. 32507392 10.1016/j.biopsych.2020.04.026 PMC7211721

[68] Dyer JAT, Kreitman N. Hopelessness, depression and suicidal intent in parasuicide. Br J Psychiatry. 1984;144(2):127–33. 6704597 10.1192/bjp.144.2.127

[69] Scocco P, Marietta P, Tonietto M, Dello Buono M, De Leo D. The role of psychopathology and suicidal intention in predicting suicide risk: A longitudinal study. Psychopathology. 2000;33(3):143–50. 10773773 10.1159/000029136

[70] Ma S, Wen Z, Sun L, Zheng Y, Zhang Y, Shi L, et al. Current situation and influencing factors for suicidal intent in patients with intentional acute pesticide poisoning. Front Public Health. 2023;11:1168176. 37089502 10.3389/fpubh.2023.1168176 PMC10117821

[71] Silver MA. Relation of depression of attempted suicide and seriousness of intent. Arch Gen Psychiatry. 1971;25(6):573. 5141377 10.1001/archpsyc.1971.01750180093015

[72] Haw C, Hawton K, Houston K, Townsend E. Correlates of relative lethality and suicidal intent among deliberate Self-Harm patients. Suicide Life-Threatening Behav. 2003;33(4):353–64. 10.1521/suli.33.4.353.25232 14695050

[73] Hatkevich C, Venta A, Sharp C. Theory of Mind and suicide ideation and attempt in adolescent inpatients. J Affect Disord. 2019;256:17–25. 31158712 10.1016/j.jad.2019.05.051

[74] Spirito A, Overholser J. The suicidal child. Child Adolesc Psychiatr Clin N Am. 2003;12(4):649–65. 14579644 10.1016/s1056-4993(03)00034-8

[75] Foran HM, Fraude I, Kubb C, Wamboldt MZ. Assessment of the Parent-Child Relationship. In: Wampler KS, McWey LM, editors. The Handbook of Systemic Family Therapy [Internet]. 1st ed. Wiley; 2020 [cited 2024 Oct 28]. pp. 35–54. Available from: https://onlinelibrary.wiley.com/doi/10.1002/9781119438519.ch35

[76] Wharff EA, Ginnis KB, Ross AM, White EM, White MT, Forbes PW. Family-Based crisis intervention with suicidal adolescents: A randomized clinical trial. Pediatr Emer Care. 2019;35(3):170–5. 10.1097/PEC.0000000000001076 28248838

[77] Hickey K, Rossetti J, Strom J, Bryant K. Issues most important to parents after their children’s suicide attempt: A pilot D Elphi study. Child Adoles Psych Nurs. 2015;28(4):157–64. 10.1111/jcap.12124 26470630

[78] Sibut R, Lachal J. Discordance in the Evaluation of Suicidal Intent Between Parents and Adolescents in Adolescence and Evolution of the Suicidal Crisis: a Mixed Study (discord-Ados) [Internet]. 2023 [cited 2024 Jun 6]. Available from: https://clinicaltrials.gov/study/NCT06036290?term=Adolescent%20Suicide,%20discord&rank=1

[79] de Kernier N. Suicide attempt during adolescence: A way of killing the infans and a quest for Individuation-Separation. Crisis. 2012;33(5):290–300. 22562857 10.1027/0227-5910/a000135

[80] Wagner BM, Aiken C, Mullaley PM, Tobin JJ. Parents’ reactions to adolescents’ suicide attempts. J Am Acad Child Adolesc Psychiatry. 2000;39(4):429–36. 10761344 10.1097/00004583-200004000-00011

[81] Cong EZ, Cai YY, Wang Y, Wu Y. Association of depression and suicidal ideation with parenting style in adolescents. Zhongguo Dang Dai Er Ke Za Zhi. 2021;23(9):938–43. 34535210 10.7499/j.issn.1008-8830.2105124 PMC8480172

[82] Weissinger GM, Evans L, Van Fossen C, Winston-Lindeboom P, Ruan‐Iu L, Rivers AS. Parent experiences during and after adolescent suicide crisis: A qualitative study. Int J Mental Health Nurs. 2023;32(3):917–28. 10.1111/inm.13137 36882964

[83] Rheinberger D, Shand F, McGillivray L, McCallum S, Boydell K. Parents of adolescents who experience suicidal Phenomena—A scoping review of their experience. IJERPH. 2023;20(13):6227. 37444075 10.3390/ijerph20136227 PMC10340647

[84] Bearss K, Taylor CA, Aman MG, Whittemore R, Lecavalier L, Miller J, et al. Using qualitative methods to guide scale development for anxiety in youth with autism spectrum disorder. Autism. 2016;20(6):663–72. 26395234 10.1177/1362361315601012

