# Supplementary material for: Characterizing suicidal intent among suicidal adolescents: a systematic review
Source: Child Adolesc Psychiatry Ment Health. 2026 Jan 2;20:17. doi: 10.1186/s13034-025-01019-8 (PMC12866032; doi:10.1186/s13034-025-01019-8)
Supplement: Supplementary file 1 — Supplementary material 1. [file 13034_2025_1019_MOESM1_ESM.docx]

#### Additional Information

# Appendix 1: PICO and keywords

| **PICO** | **Keywords** |
| --- | --- |
| Population  Patient  Problem | Adolescents  Young  Teen  Suicidal intent  Suicide intent |
| Intervention or Exposure | Suicide attempt |
| Comparison | Characteristics |
| Outcomes |  |

# Appendix 2: research stages

KEY WORDS

Suicidal intent

Suicide intent

Young

Adolescent

Suicidal attempter

Suicide attempt

Characteristic

PubMed

Search 1

**((((((Suicidal intent) AND (suicide intent)) AND (Young)) AND (Adolescent)) AND (suicide attempter)) AND (Suicidal attempter)) AND (characteristic)** Filters: **Adolescent: 13-18 years, Child: 6-12 years**

**111 results**

**url:**

https://pubmed.ncbi.nlm.nih.gov/?term=%28%28%28%28%28%28Suicidal+intent%29+AND+%28Suicid+intent%29%29+AND+%28Young%29%29+AND+%28Adolescent%29%29+AND+%28Suicid+attempter%29%29+AND+%28Suicidal+attempter%29%29+AND+%28Charasteristic%29&filter=age.adolescent&filter=age.child&sort=relevance

Search 2

**Etape 1:**

**((Suicide intent[MeSH Terms]) AND (Adolescent[MeSH Terms])) AND (Suicidal intent[MeSH Terms])**

**151 results**

**url:**

[**https://pubmed.ncbi.nlm.nih.gov/?term=%28%28Suicide+intent%5BMeSH+Terms%5D%29+AND+%28Adolescent%5BMeSH+Terms%5D%29%29+AND+%28Suicidal+intent%5BMeSH+Terms%5D%29&sort**](https://pubmed.ncbi.nlm.nih.gov/?term=%28%28Suicide+intent%5BMeSH+Terms%5D%29+AND+%28Adolescent%5BMeSH+Terms%5D%29%29+AND+%28Suicidal+intent%5BMeSH+Terms%5D%29&sort)**=**

**Etape 2:**

**((Suicide intent[Title/Abstract]) AND (Adolescent[Title/Abstract])) AND (Suicidal intent[Title/Abstract])**

**7 results**

**url:** <https://pubmed.ncbi.nlm.nih.gov/?term=%28%28Suicide+intent%5BTitle%2FAbstract%5D%29+AND+%28Adolescent%5BTitle%2FAbstract%5D%29%29+AND+%28Suicidal+intent%5BTitle%2FAbstract%5D%29&sort>**=**

Search 3

**(((Suicidal intent) AND (Suicide intent)) AND (Adolescent)) AND (Suicide attempt)**

**869 Results**

**url:**

<https://pubmed.ncbi.nlm.nih.gov/?term=%28%28%28Suicidal+intent%29+AND+%28Suicide+intent%29%29+AND+%28Adolescent%29%29+AND+%28Suicide+attempt%29&sort>**=**

Search 4

**(((Suicidal intent) AND (Suicide intent)) AND (Adolescent)) AND (Suicide attempt)**

**Filtres :** *Filters applied: Child: birth-18 years, Child: 6-12 years, Adolescent: 13-18 years*

833 Results

url:

**https://pubmed.ncbi.nlm.nih.gov/?term=%28%28%28Suicidal+intent%29+AND+%28Suicide+intent%29%29+AND+%28Adolescent%29%29+AND+%28Suicide+attempt%29&filter=age.allchild&filter=age.child&filter=age.adolescent**

Search 5

(((Suicidal intent[MeSH Terms]) AND (Suicide intent[MeSH Terms])) AND (Adolescent[MeSH Terms])) AND (Suicide attempt[MeSH Terms])

82 Results (with and without filters)

url:

<https://pubmed.ncbi.nlm.nih.gov/?term=%28%28%28Suicidal+intent%5BMeSH+Terms%5D%29+AND+%28Suicide+intent%5BMeSH+Terms%5D%29%29+AND+%28Adolescent%5BMeSH+Terms%5D%29%29+AND+%28Suicide+attempt%5BMeSH+Terms%5D%29&sort>=

Elsevier Masson

“Suicidal intent”+”Suicide intent”+adolescent+”Suicide attempt” in all text
Publication period: All years and in all site products

20 results in English

25 results in English

url:

<https://www-em-premium-com.ezproxy.uca.fr/recherche/resultats>

COCHRANE

(Suicidal intent) AND(Suicide intent) AND (adolescent) AND (Suicide attempt) in All Text - (Word variations have been searched)

27 results

Url:

https://www-cochranelibrary-com.ezproxy.uca.fr/advanced-search?q=%22Suicidal%20intent%22%2B%22Suicide%20intent%22%2Badolescent%2B%22Suicide%20attempt%22&t=6

CAIRN

(Suicide intent)+Adolescent+(Suicidal intent)

21 results

url:

https://www-cairn-info.ezproxy.uca.fr/resultats_recherche.php

## Table of different stages of research

|  | PubMed | EM | | Cairn | | Cochrane | | Science Direct |
| --- | --- | --- | --- | --- | --- | --- | --- | --- |
| Filters |  | | | | | | |  |
| ∅ | (((Suicidal intent) AND (Suicide intent)) AND (Adolescent)) AND (Suicide attempt)  869 | "Suicidal intent"+"Suicide intent"+adolescent+"Suicide attempt"  20 french  25 English | | (Suicide intent)+Adolescent+(Suicidal intent)+(suicide attempt)  15 | | (Suicidal intent) AND(Suicide intent) AND (adolescen) AND (Suicide attempt) in All Text    27 | | "suicide intent" and adolescent  407 |
| Link PubMed | https://pubmed.ncbi.nlm.nih.gov/?term=%28%28%28Suicidal+intent%29+AND+%28Suicide+intent%29%29+AND+%28Adolescent%29%29+AND+%28Suicide+attempt%29 | | | | | | |  |
| Link EM | https://www-em-premium-com.ezproxy.uca.fr/recherche/resultats | | | | | | |  |
| Link Cairn | https://www-cairn-info.ezproxy.uca.fr/resultats_recherche.php?searchTerm=%28Suicide+intent%29%2BAdolescent%2B%28Suicidal+intent%29%2B%28suicide+attempt%29&searchTermStored=all | | | | | | |  |
| Link Science direct | https://www-sciencedirect-com.ezproxy.uca.fr/search?qs=%22suicide%20intent%22%20and%20adolescent | | | | | | |  |
| Link Cochrane | https://www-cochranelibrary-com.ezproxy.uca.fr/search | | | | | | |  |
| FILTER | PubMed | | em | | cairn | | cochrane | Sci Direct |
| *Filters applied: Child: birth-18 years, Child: 6-12 years, Adolescent: 13-18 years* | 833 | | No population age filters applicable  20 French  25 English | | No population age filters applicable  15 | | No population age filters applicable  27 | No population age filters applicable  407 |
| FILTER |  | |  | |  | |  |  |
|  | Full text available  733 | | Not applicable  20  25 | | Full text available  7 | | Not applicable  27 | 407 |
| Filters | Pub | | EM | | cairn | | Cochrane | Science Direct |
| Mesh | MESH TERM+ Full text, Child: birth-18 years, Child: 6-12 years, Adolescent: 13-18 years  (((Suicidal intent[MeSH Terms]) AND (Suicide intent[MeSH Terms])) AND (Adolescent[MeSH Terms])) AND (Suicide attempt[MeSH Terms])  82 | | 56 English  Cf url  "Suicidal intent"+"Suicide intent"+adolescent+"Suicide attempt"" in Key Words  Publication Period : all years  Pediatric Clinics of North America  - Pediatric Neurology  - Soins Pédiatrie/Puériculture  - Soins Psychiatrie  - TCC chez l'enfant et l'adolescent  - Urgences psychiatriques | | Keywords 🡪 0 | | Removal of suicide attempt  Filter: Title Abstract Keyword  (Suicidal intent) AND(Suicide intent) AND (adolescent) in Title Abstract Keyword  8 | Title, abstract or author-specified keywords  11 |
| Link pub | https://pubmed.ncbi.nlm.nih.gov/?term=%28%28%28Suicidal+intent%5BMeSH+Terms%5D%29+AND+%28Suicide+intent%5BMeSH+Terms%5D%29%29+AND+%28Adolescent%5BMeSH+Terms%5D%29%29+AND+%28Suicide+attempt%5BMeSH+Terms%5D%29&filter=simsearch3.fft&filter=age.allchild&filter=age.child&filter=age.adolescent | | | | | | |  |
| Em | https://www-em-premium-com.ezproxy.uca.fr/recherche/resultats | | | | | | |  |
| Cochrane | https://www-cochranelibrary-com.ezproxy.uca.fr/advanced-search?q=(Suicidal%20intent)%20AND(Suicide%20intent)%20AND%20(adolescen)%20AND%20(Suicide%20attempt)&t=6 | | | | | | |  |
| Link SDirect | https://www-sciencedirect-com.ezproxy.uca.fr/search?qs=%22suicide%20intent%22%20and%20adolescent&tak=%22suicide%20intent%22%20and%20adolescent | | | | | | |  |

# Appendix 3: NIH Quality Assessment Tool

NIH Quality Assessment Tool for Observational Cohort and Cross-Sectional Studies

| CR | Q1 | Q2 | Q3 | Q4 | Q5 | Q6 | Q7 | Q8 | Q9 | Q10 | Q11 | Q12 | Q13 | Q14 |
| --- | --- | --- | --- | --- | --- | --- | --- | --- | --- | --- | --- | --- | --- | --- |
| 1 | V | V | V | V | V | X | NA | NA | V | X | V | NA | NA | V |
| 2 | V | V | V | V | X | NA | NA | NA | NA | NA | V | NA | V | NA |
| 3 | V | V | V | V | V | NA | NA | NA | NA | NA | V | NA | V | NA |
| 4 | V | V | V | V | V | NA | NA | NA | NA | NA | V | NA | V | NA |
| 5 | V | V | V | V | V | NA | NA | NA | NA | NA | V | NA | V | NA |
| 6 | V | V | NR | V | X | NA | NA | NA | NA | NA | V | NA | V | NA |
| 7 | V | V | NR | V | V | NA | NA | NA | NA | NA | V | NA | V | NA |
| 8 | V | V | V | V | X | X | V | NA | V | NA | V | V | V | V |

| RS | Q1 | Q2 | Q3 | Q4 | Q5 | Q6 | Q7 | Q8 | Q9 | Q10 | Q11 | Q12 | Q13 | Q14 |
| --- | --- | --- | --- | --- | --- | --- | --- | --- | --- | --- | --- | --- | --- | --- |
| 1 | V | V | V | V | V | V | NA | NA | V | X | V | NA | NA | V |
| 2 | V | V | V | V | X | NA | NA | NA | NA | NA | V | NA | V | NA |
| 3 | V | V | V | V | V | NA | NA | NA | NA | NA | V | NA | V | NA |
| 4 | V | V | V | V | V | NA | NA | NA | NA | NA | V | NA | V | NA |
| 5 | V | V | V | V | V | NA | NA | NA | NA | NA | NA | NA | V | NA |
| 6 | V | V | NR | V | X | NA | NA | NA | NA | NA | V | NA | V | NA |
| 7 | V | V | NR | V | V | NA | NA | NA | NA | NA | V | NA | V | NA |
| 8 | V | V | V | V | X | X | V | NA | V | NA | V | V | V | V |

### Number related to the study

|  | Study | RS | CR |
| --- | --- | --- | --- |
| 1 | Age- and Sex-Related Risk Factors for Adolescent Suicide – Brent, 1999 | Good | Good |
| 2 | Life problems and deliberate self-harm: Associations with gender, age, suicidal intent and psychiatric and personality disorder – Haw et Hawking, 2008 | Good | Good |
| 3 | Suicidal Intent and Method of Self-Harm: A Large-scale Study of Self-Harm Patients Presenting to a General Hospital – Haw, 2015 | Good | Good |
| 4 | Characteristics of high intent suicide attempters admitted to a general hospital – Kumar, 2006 | Fair | Fair |
| 5 | Motives and Suicide Intent Underlying Hospital Treated Deliberate Self-Harm and Their Association with Repetition - McAuliffe, 2007 | Fair | Good |
| 6 | Clinical Components of Suicidal Intent in Adolescent Overdose – Kingsbury, 1993 | Poor | Fair |
| 7 | Hopelessness, Depression, and Suicidal Intent among Adolescent Suicide Attempters – Rotheram-Borus, 1988 | Poor | Poor |
| 8 | Adolescent US Poison Center Exposure Calls During the COVID-19 Pandemic – Wang, 2022 | Good | Good |

**Note**: Global quality rating according to NIH Quality Assessment Tool guidelines — Good, Fair, or Poor based on the number and relevance of positive items (Q1–Q14).

RS: Romain SIBUT

CR: Clara ROBERT

Q: NIH Item number

V: yes

X: no

NA/NR: not applicable/ not reported

Quality Assessment of Case-Control Studies

| RS | Q1 | Q2 | Q3 | Q4 | Q5 | Q6 | Q7 | Q8 | Q9 | Q10 | Q11 | Q12 |
| --- | --- | --- | --- | --- | --- | --- | --- | --- | --- | --- | --- | --- |
| 1 | V | V | X | V | V | V | V | X | V | V | X | V |
| 2 | V | V | X | V | V | V | V | X | V | V | V | V |
| CR | Q1 | Q2 | Q3 | Q4 | Q5 | Q6 | Q7 | Q8 | Q9 | Q10 | Q11 | Q12 |
| 1 | V | V | X | V | V | V | V | X | V | V | X | V |
| 2 | V | V | X | V | V | V | V | X | V | V | V | V |

### Number related to the study

|  | Study | RS | CR |
| --- | --- | --- | --- |
| 1 | Association of Socioeconomic Status, Problem Behaviors, and Disordered Eating in Mexican Adolescents: Results of the Mexican National Health and Nutrition Survey 2006 – Palma-Coca, 2011 | Good | Good |
| 2 | Adolescents hospitalized with deliberate self-harm: the significance of an intention to die – Groholt, 2000 | Good | Good |

**Note**: Global quality rating according to NIH Quality Assessment Tool guidelines — Good, Fair, or Poor based on the number and relevance of positive items (Q1–Q12).

RS: Romain SIBUT

CR: Clara ROBERT

Q: NIH Item number

V: yes

X: no

NA/NR: not applicable/ not reported

Quality Assessment Tool for Before-After (Pre-Post) Studies With No Control Group

| RS | Q1 | Q2 | Q3 | Q4 | Q5 | Q6 | Q7 | Q8 | Q9 | Q10 | Q11 | Q12 |
| --- | --- | --- | --- | --- | --- | --- | --- | --- | --- | --- | --- | --- |
| 1 | V | V | V | X | V | V | V | X | V | V | V | NA |
| CR | Q1 | Q2 | Q3 | Q4 | Q5 | Q6 | Q7 | Q8 | Q9 | Q10 | Q11 | Q12 |
| 1 | V | V | V | V | V | V | V | X | V | V | V | NA |

### Number related to the study

|  | Study | RS | CR |
| --- | --- | --- | --- |
| 1 | Level of suicidal intent predicts overall mortality and suicide after attempted suicide: a 12-year follow-up study – Suominen, 2004 | Good | Good |

**Note**: Global quality rating according to NIH Quality Assessment Tool guidelines — Good, Fair, or Poor based on the number and relevance of positive items (Q1–Q12).

RS: Romain SIBUT

CR: Clara ROBERT

Q: NIH Item number

V: yes

X: no

NA/NR: not applicable/ not reported
